# Germline Transmission of an Embryonic Stem Cell Line Derived from BALB/c Cataract Mice

**DOI:** 10.1371/journal.pone.0090707

**Published:** 2014-03-04

**Authors:** Xinrong Peng, Tao Liu, Chuanyin Shi, Liqing Zhang, Ying Wang, Wuyang Zhao, Lihua Jiang, Mengchao Wu, Yong Zhang, Qijun Qian

**Affiliations:** 1 College of Veterinary Medicine, Northwest Agriculture and Forestry University, ShanXi, China; 2 Eastern Hepatobiliary Surgical Hospital, The Second Military Medical University, Shanghai, China; 3 Xinyuan Institute of Medicine and Biotechnology, College of Life Science, Zhejiang Sci-Tech University, Hangzhou, Zhejiang, China; Baylor College of Medicine, United States of America

## Abstract

Mice embryonic stem (ES) cells have enabled the generation of mouse strains with defined mutation(s) in their genome for putative disease loci analysis. In the study of cataract, the complex genetic background of this disease and lack of long-term self-renewal ES cells have hampered the functional researches of cataract-related genes. In this study, we aimed to establish ES cells from inherited cataract mice (BALB/C^Cat/Cat^). Embryos of cataract mice were cultured in chemical-defined N2B27 medium with the presence of two small molecules PD0325901 and CHIR99021 (2i) and an ES cell line (named EH-BES) was successfully established. EH-BES showed long-term self-renewal in 2i medium and maintained capacity of germline transmission. Most importantly, the produced chimera and offspring developed congenital cataract as well. Flow cytometry assay revealed that EH-BES are homogeneous in expression of Oct4 and Rex1in 2i medium, which may account for their self-renewal ability. With long-term self-renewal ability and germline-competent, EH-BES cell line can facilitate genetic and functional researches of cataract-related genes and better address mechanisms of cataract.

## Introduction

Cataract is the most common cause of blindness and the development of cataract can be triggered by a variety of reasons including aging, trauma, radiation, etc. Another strong component in cataract is genetic abnormalities and approximately half of congenital cataract cases may have a genetic cause [1]. Many loci were found to be responsible for human inherited cataracts and over 26 of them were associated with causative mutations in specific genes [2]–[4]. Application of small molecules targeting cataract-related genes is a potentially feasible non-surgical approach for cataract prevention [5]. However, challenges for understanding the genetic mechanism of congenital cataract remain due to the high density of sequence variation within candidate loci.

For efficient loci analysis via gene targeting, embryonic stem (ES) cells are commonly employed. Since inherited cataract mice are valuable disease model of human, their ES cells are ideal materials for genetic studies of cataract. Mice ES cells were first derived from 129 mice strain [6], [7]. However, ES cells from other strains such as BALB/C mice are refractory to self-renewal under standard culture conditions and eventually achieved using conditioned medium on a layer of 5637 bladder carcinoma feeder cells [8]. Later emerged chemical-defined 2i medium [9] has enabled the derivation of ES cells from C57BL/6 mice, Kunming mice, and for the first time from rat [10]–[12]. So far, it has not been proved whether ES cells from BALB/C hereditary cataract mice can maintain self-renewal and germline transmission ability in 2i medium..

In this study, we established an ES cell line (named EH-BES) from BALB/C hereditary cataract mice (BALB/C^Cat/Cat^) using the 2i medium. The BALB/C strain has advantages in studying genetic diseases such as diabetes [13] and cataract [14]. EH-BES cells established here maintained long-term self-renewal and exhibited efficient germline transmission ability, which can facilitate researches of cataract-related genes and the involved mechanisms. Flow cytometry assay indicated that EH-BES cells are rather homogeneous in 2i medium in expression of two pluripotency markers: Oct4 and Rex1[15], which may account for their long-term self-renewal abilities.

## Materials and Methods

### Derivation and propagation of EH-BES

All animal experiments were approved by the Second Military Medical University Committee on Animal Care (EC11-055) and performed under the National Institutes of Health Guidelines on the Use of Laboratory Animals. All mice were purchased from Shanghai Experimental Animal Center, Chinese Academy of Sciences and kept at 22°C on a 12 h light-dark cycle with free access to food and water. Mice were sacrificed by cervical dislocation. Embryonic (E) day 3.5 embryos were obtained from BALB/C^Cat/Cat^ mice and cultured in 2i medium on gelatin-coated plate with culture medium changed by half every day. The derived ES cells (named EH-BES) were passaged using 0.05% trypsin-EDTA (Invitrogen, 25300054) with a split ratio of 1 to 10. EH-BES cells were maintained in chemical-defined 2i medium on gelatin-coated plastics. For treatment experiment, the cells were separately cultured in N2B27 medium, serum medium, and 2i medium. The chemical-defined N2B27 medium was prepared as previously described [16] and the finalized 2i medium contained the addition of 1 µM PD0325901 (Stemgent, 04000610) and 3 µM CHIR99021 (Stemgent, 04000410) to N2B27 medium [9], [17]. The serum medium was prepared as previously described which contains 10 ng/ml leukemia inhibitory factor (LIF) (Millipore, LIF2010) and 15% fetal bovine serum (FBS) (Gibco, 10100147) [18].

### Differentiation of EH-BES

Production of embryoid bodys (EBs) was performed as previously described [19]. Briefly, EH-BES cells were removed onto bacterial grade petri dishes and cultured in the serum medium not containing LIF. Expression of markers for three germ layers was then examined by RNA analysis on differentiation day 0, 7 and 10. For teratoma formation, the EH-BES cells were injected subcutaneously into nude mice (2 × 10^6^ cells per mouse). The formed teratoma was collected and fixed in 4% paraformaldehyde (Sigma-Aldrich, 30525894) and stained with haematoxylin and eosin (H&E) following standard procedures.

### Immunostaining and alkaline phosphatase staining assay

Immunostaining assay was performed according to standard protocols. Briefly, the cells were fixed in 4% paraformaldehyde and washed with phosphate-buffered saline (PBS) (Invitrogen, 10010023) and then incubated in PBS containing 0.2% Triton X-100 (Sigma-Aldrich, 9002931) and 0.3% bovine serum albumin (Invitrogen, 11020021). Afterwards, the cells were incubated with primary antibodies including: goat anti-Oct4 (Abcam, ab27985), mouse anti-Nanog (Santa Cruz, sc-374001), mouse anti-Sox2 (Abcam, ab75485), goat anti-GATA4 (Santa Cruz, sc-1237), mouse anti-tyrosine hydroxylase (TH) (Abcam, ab6211), and goat anti- alpha fetoprotein (AFP) (Santa Cruz, sc-8108). The cells were then washed and incubated with goat anti-mouse IgG or rabbit-anti-goat IgG (Invitrogen). A concentration of 0.5 µg/ml diamino phenyl indole (DAPI) (Sigma, 28718903) was used for nuclei staining. Images were visualized using Nikon Ti inverted fluorescence microscope. Alkaline phosphatase (AP) staining was performed using the AP detection kit (Millipore, SCR004).

### RT-PCR analysis

EH-BES cells at differentiation day 0, 7 and 10 were collected and total RNA was extracted using RNeasy mini kit (QIAGEN, 74104). Then cDNA was synthesized from total RNA (1 µg) by using QuantiTect-Reverse-Transcription kit (QIAGEN, 205313). Markers such as Oct4, Nestin, GATA4, AFP, FLK1 and Sox17 were evaluated to analyze the extent of cell differentiation, while Eif4g2 was used as loading control. The complete primer information was listed in Table S1.

### Chimera generation

To evaluate karyotype of EH-BES, analysis was performed as previously described [20]. Then EH-BES cells were aggregated with zona-free embryos to form chimeras. The recipient embryos were recovered from the oviduct of 129Sv females at embryonic day 2.5 and incubated with acidified Tyrode’s solution to remove zona pellucida. Clumps of EH-BES cells (8 to 15 cells) were collected and cultured with the zona-free embryos in KSOM medium (Millipore, MR-121-D) to obtain aggregated embryos. The aggregated embryos were then transferred into pseudo-pregnant recipient females. Chimeras were identified by coat color and germline transmission was tested by mating the chimeras with normal BALB/C mice.

### Flow cytometry assay

EH-BES cells were separately cultured in N2B27 medium, serum medium, and 2i medium for 7 days. Then cells from each group were fixed and permeabilized using BD Cytofix/Cytoperm solution (BD Biosciences) according to manufacturer’s instruction. Flow cytometry assay was performed according to a standard protocol. Briefly, the cells were incubated with mouse anti-Rex1 (Abcam, ab175429) and goat anti-Oct4 (Abcam, ab27985) for 60 min at 4°C. Then the cells were incubated with Alexa Fluor 488 goat anti-mouse IgG (Invitrogen, A-11001) or rabbit-anti-goat IgG (Invitrogen, A-11078). Flow cytometry assay was carried out using FC500 flow cytometer (Beckman Coulter). Statistical analysis was performed by using SPSS 11.0 (IBM Corp. USA). The experiments were separately repeated for 3 times and results were presented as means and standard deviation (SD).

## Results and Discussion

### EH-BES cells maintained self-renewal and pluripotency in 2i medium

**Figure 1:**
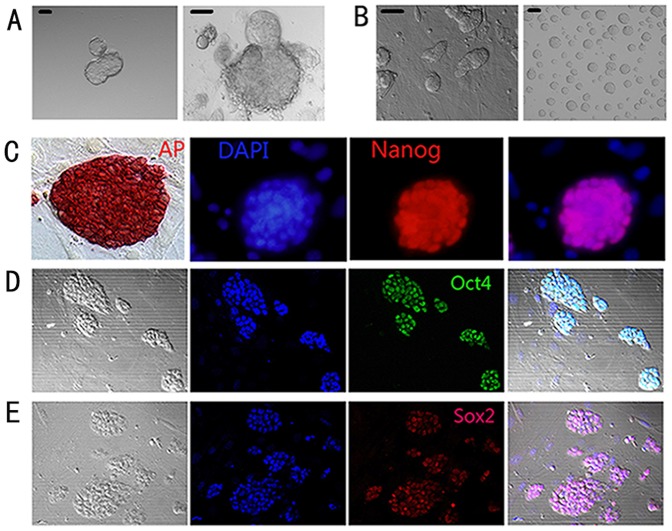
Establishment of EH-BES cell line in 2i medium. (A) Embryos from BALB/C^Cat/Cat^ mice were separately seeded in 2i medium on gelatin-coated plastics and ES cells-like colony emerged in 3 days. (B) EH-BES cell line was established and maintained with or without feeders. (C) EH-BES cells were AP-staining positive (left); EH-BES cells showed Nanog-expression by immunostaining analysis (right). (D) EH-BES cells showed Oct4-expression. (E) EH-BES cells showed Sox2-expression. DAPI stain indicates nuclei in blue. Scale bars stand for 100 µm.

To derive ES cells, embryos from BALB/C^Cat/Cat^ mice were cultured in 2i medium with the presence of 1 µM PD0325901 and 3 µM CHIR99021 as previously described [9] and results showed that undifferentiated ES-like colonies appeared in 3 days on gelatin-coated plastics (Fig. 1A). These colonies had been propagated to passage 70 in 2i medium and still maintained self-renewal, from which an ES cell line (named EH-BES, deposited at China Center for Type Culture Collection with access number C201217 on February 2012) was successfully established (Fig. 1B). Previous studies suggested that ES cells should be cultured on feeder layer in order to obtain the necessary factors such as LIF [7]. In this study, we showed that EH-BES cells could maintain long-term self-renewal in chemical-defined medium in the absence of LIF and feeder (Fig.1B), indicating that LIF is unnecessary for maintaining self-renewal of EH-BES cell line. We then examined the pluripotency of EH-BES cell line using AP-staining method since undifferentiated pluripotent stem cells have elevated levels of AP-expression on their cell membrane [15] and confirmed the pluripotency of EH-BES cells with positive AP-staining (Fig. 1C). Immunostaining assays showed that EH-BES cells had expression of Nanog (Fig. 1C), which plays a crucial role in the maintenance of pluripotency [21]. In addition, immunostaining assays also confirmed that EH-BES cells showed Oct4 (Fig. 1D) and Sox2 (Fig. 1E) expression, which are considered to be essential in the regulation of stem cell pluripotency [15], [22]. Taken together, the results showed that EH-BES cells can maintain self-renewal and pluripotency in chemical-defined culture condition.

### EH-BES differentiated into cells and tissues from all three germ layers

**Figure 2:**
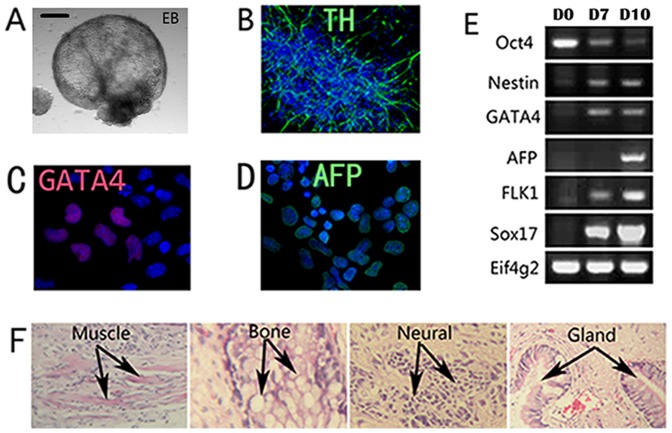
EH-BES differentiated into cells and tissues from all three germ layers. (A) EH-BES cells formed EBs after 7 days of differentiation and image was taken at day 7. Immunostaining assay of differentiation day 10 cultures for TH (ectoderm) (B), GATA-4 (mesoderm) (C), and AFP (endoderm) (D). (E) RT-PCR analysis of differentiation cultures on day 0 (D0), day7 (D7), and day 10 (D10) showed down-regulated Oct4-expression and up-regulated expression of Nestin, GATA-4, AFP, FLk1, and Sox17. (F) EH-BES cells-formed teratoma contained tissues and cells from all three germ layers including muscle (mesoderm), bone (mesoderm), neural tissues (ectoderm), and gland tissues (endoderm). Scale bars stand for 100μm.

To evaluate the differentiation ability of EH-BES cells, we cultured the cells in serum medium (not containing LIF) to produce EBs as previously described[19]. Under this condition, EH-BES cells grew in suspension and formed EBs within 7 days (Fig. 2A). Within EBs, ES cells’ differentiation proceeded at a schedule similar to that in the embryo [19]. After 10 day differentiation, immunostaining assays indicated that the produced EBs showed positive expression of markers for ectoderm (TH) (Fig. 2B), mesoderm (GATA-4) (Fig. 2C), and endoderm (AFP) (Fig. 2D). RT-PCR analysis confirmed that the differentiated cells showed rapid reduction of Oct4-expression on differentiation day 7 and 10 (Fig. 2E). Along with the reduction of Oct4, expression of several lineage-specific molecular markers such as Nestin (ectoderm), GATA4, FLk1 (mesoderm), AFP, and Sox17 (endoderm) was up-regulated (Fig. 2E). For a functional assessment of the pluripotency, teratoma formation was also performed to confirm the presence of three germ layers structures. After injection of EH-BES into nude mice, these cells can form teratoma in nude mice within 3 months. Moreover, the formed tumors contained tissues from all three germ layers, such as muscle and bone (mesoderm), gland (endoderm), and neural tissues (ectoderm) (Fig. 2F).

### EH-BES can form chimeras and transmit through the germline

**Figure 3:**
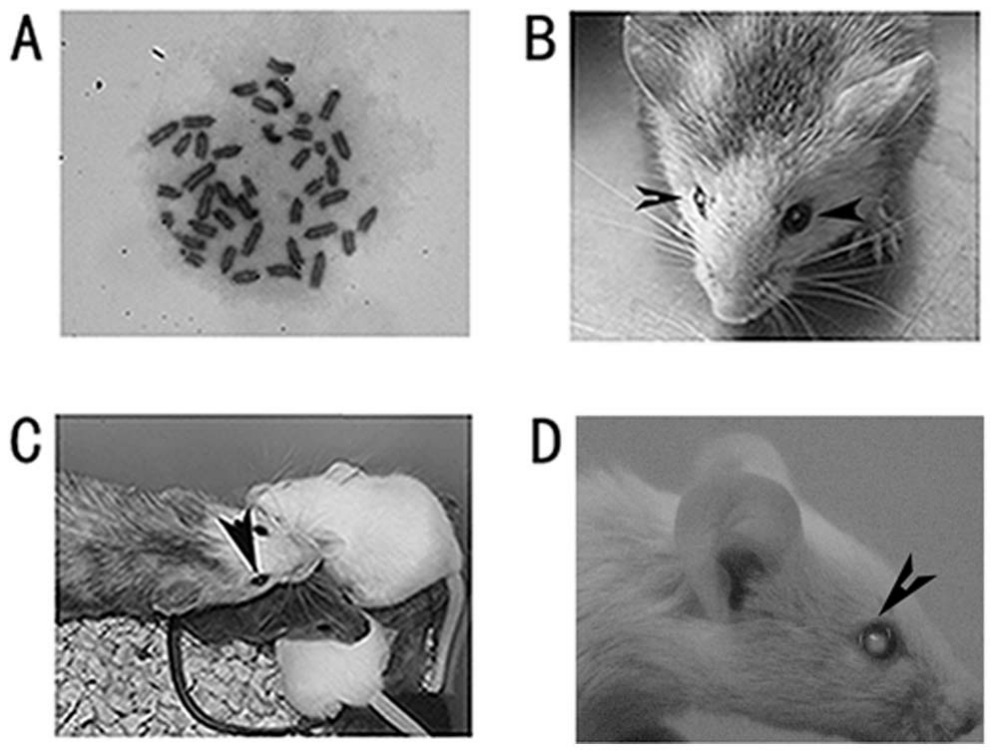
EH-BES had normal karyotype and produced chimeras. (A) EH-BES had normal karyotype (40 chromosomes). (B) The female chimera showed chimeric coat color and cataract development (indicated by arrow). (C) The female chimera (indicated by arrow) produced four offspring after mated with normal BALB/C mouse, of which two offspring showed white coat color. (D) The white offspring showed cataract development (indicated by arrow).

To confirm that EH-BES are normal cells, karyotype of EH-BES was analyzed and results showed that EH-BES cell line still maintained normal karyotype at passages 65 (Fig. 3A). We then evaluated the germline transmission ability of EH-BES since germline contribution assay is the gold standard for the identity of genuine ES cells. We aggregated EH-BES cells into 129Sv blastocysts and transferred 38 aggregated blastocysts into five pseudo-pregnant mice. Three chimeric mice (two male and one female) were obtained with coat color indicative of the presence of EH-BES cell and opacification of lens was observed in the female chimera (Fig. 3B). The female chimera was then mated with normal BALB/C mice and produced four offspring including two white coat color and two agouti coat color (Fig. 3C). Both white-coat-colored offspring showed cataract development (Fig. 3D). These results confirmed that EH-BES cells maintained the capacity for incorporation into the developing embryo and can transmit through the germline. BALB/C strain is the most thoroughly characterized and widely applied mouse strain and the many advantages of BALB/C over other strains such as 129 make it suitable for disease model and genetical studies [8]. However, compared with 129 mice ES cells, BABL/C mice ES cells are refractory to self-renewal under standard conditions, which hampered their applications in gene targeting. In the dissection of cataract-linked loci and their associated roles, the lack of ES cells of BALB/C hereditary cataract mice has limited such analysis. Our results showed that EH-BES cells can maintain self-renewal and germline transmission ability in chemical-defined 2i medium, which can provide an efficient platform for the loci analysis of cataract development. Later, it will be of interest to inactivate cataract-related genes in EH-BES cell line through gene manipulation, which can facilitate the generation of new transgenics and application of small molecules targeting cataract-related genes.

### EH-BES cells in 2i medium exhibited homogeneity in expression of pluripotency markers

**Figure 4:**
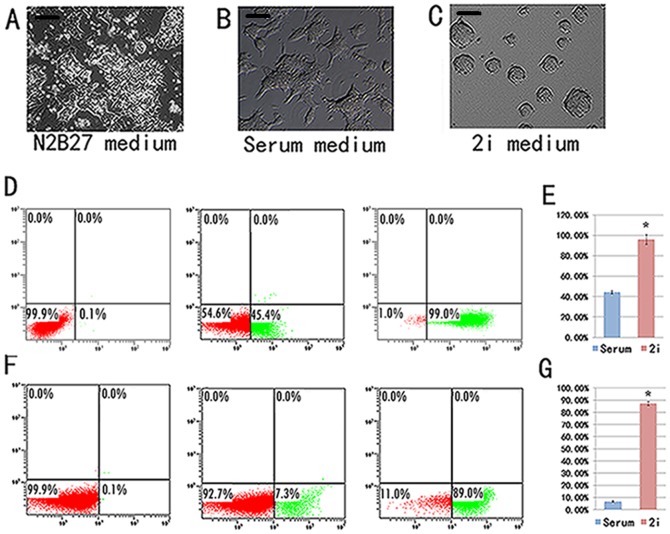
After 2 passages, EH-BES cells exhibited different morphology in different culture medium. (A) EH-BES cells differentiated in N2B27 medium. (B) EH-BES cells cultured in serum medium were heterogeneous in morphology. (C) EH-BES formed tightly-packed undifferentiated colonies in 2i medium. (D) Cells in N2B27 medium were Oct4-negative by flow cytometry assay (left); cells in serum medium were heterogeneous in expression of Oct4 (middle); cells in 2i medium were homogenous in Oct4-expression (right). (E) Cells in 2i medium showed significantly higher percentage of Oct4-positive cells compared with those in serum medium (* indicates p<0.001). (F) EH-BES cells totally lost Rex1-expression in N2B27 medium (left); EH-BES cells were heterogeneous in Rex1-expression in serum medium (middle); EH-BES cells were rather homogeneous in Rex1-expression in 2i medium (right). (G) EH-BES cells in 2i medium showed significantly higher percentage of Rex1-positive cells compared with those in serum medium (* indicates p<0.001). Scale bars stand for 100μm.

To further examine the molecular mechanism involved in the self-renewal of EH-BES cells, we separately cultured the cells in N2B27 medium, serum medium, and 2i medium for two passages. The results showed that EH-BES cells differentiated and lost self-renewal ability in N2B27 medium (Fig. 4A), while EH-BES cells were heterogeneous in morphology and produced differentiated colony in serum medium (Fig. 4B). In contrast, EH-BES cells formed tightly-packed colony and maintained long-term self-renewal in 2i medium (Fig. 4C). Flow cytometry assay confirmed that N2B27-cultured EH-BES cells completely lost Oct4-expression, whereas 2i medium yielded rather homogenous Oct4-positive cells (95.9%±4.8%) as compared with serum medium (44.1%±1.4%) (Fig. 4D, E). EH-BES totally lost Rex1 expression in N2B27 medium, while EH-BES in 2i medium showed significantly higher percentage of Rex1-positive cells (87.4%±1.7%) compared to those cultured in serum medium (6.87%±0.6%) (Fig. 4F, G). Rex1 (also known as Zfp42) is strongly expressed in the inner cell mass (ICM) [23] and can be used to distinguish the epiblast and primitive ectoderm (PrE) from the ICM [24]. Previous studies reveal that ES cells cultured in serum medium contain subpopulations corresponding to ICM, epiblast, and PrE, in which Rex1-negative and Oct4-positive subpopulations show poor ability to contribute to chimera formation [24] and may lose self-renewal capacity [25]. Differentiation of ES cells involves auto-inductive stimulation of mitogen-activated protein kinase (ERK/MAPK) signaling by fibroblast growth factor-4 (FGF4) [26], [27]. PD0325901 is a small molecule inhibitor of ERK [28] and CHIR99021 is a highly selective small molecule inhibitor of glycogen synthase kinase-3 (GSK-3) [29]. Report shows that a higher dose of PD0325901 (2 µM) can totally eliminate activation of ERK and cause growth arrest, while addition of CHIR99021 restores viability and allows efficient expansion of ES cells in the near absence of ERK signaling [9]. Our study revealed that a higher dose of PD0325901 (2 µM) combined with CHIR99021 (3 µM) showed no significant effect on propagation of EH-BES cell line but hamper germline transmission of the cells (data not shown). These results showed that 2i medium facilitated homogeneous expression of Rex1 and Oct4 in EH-BES cell line and then promoted self-renewal and germline transmission of the cells.

## Conclusion

We successfully established an embryonic stem cell line (EH-BES) from inherited cataract BALB/C mice (BALB/C^Cat/Cat^). EH-BES cell line maintained long-term self-renewal ability in chemical-defined culture condition, showed homogenous expression of pluripotency markers, exhibited efficient germline transmission, and produced offsprings with cataract developed, which can promote the dissection of cataract-linked loci and their associated roles in cataract development via gene targeting.

## Supporting Information

Table S1 **Primers information for reverse transcription PCR.**
(DOC)
