# Supplementary material for: Germline Transmission of an Embryonic Stem Cell Line Derived from BALB/c Cataract Mice
Source: PLoS One. 2014 Mar 4;9(3):e90707. doi: 10.1371/journal.pone.0090707 (PMC3942454; doi:10.1371/journal.pone.0090707)
Supplement: Table S1 — Primers information for reverse transcription PCR. (DOC) [file pone.0090707.s001.doc]

**Table S1**

| Gene | oriention | Primers F(top), R(bottom) |
| --- | --- | --- |
| Nestin | sense | GCCAAGGTGGGGGTTCTGGC |
|  | antisense | TGGGCAGCAACTGGCACACC |
| GATA4 | sense | GCTGCCACCCGAGGTGGTTC |
|  | antisense | CCCTGGCGCCTTCATGCACA |
| Sox17 | sense | GGGGCCCATGTGCGGAGACAT |
|  | antisense | AGACGAACGCAAGCGGTTGGC |
| FLK1 | sense | GTGCGGGAGGGCGGACTCTA |
|  | antisense | GACTCACGGAGATCGCGCCG |
| AFP | sense | GCCATTCCCTCACCACAGCCG |
|  | antisense | GTGCTGCCAGCTCAGCGAGG |
| OCT4 | sense | TCTTTCCACCAGGCCCCCGGCTC |
|  | antisense | TGCGGGCGGACATGGGGAGATCC |
| Eif4g2 | sense | ATTCTTCGTTGTCAAGCCGCCAAAGTG |
|  | antisense | AGTTGTTTGCTGCGGAGTTGTCATCTCG |
